# Gamma-phage lysin PlyG sequence-based synthetic peptides coupled with Qdot-nanocrystals are useful for developing detection methods for *Bacillus anthracis *by using its surrogates, *B. anthracis-Sterne *and *B. cereus-4342*

**DOI:** 10.1186/1472-6750-9-67

**Published:** 2009-07-22

**Authors:** Shilpakala Sainathrao, Ketha V Krishna Mohan, Chintamani Atreya

**Affiliations:** 1 Section of Cell biology, Laboratory of Cellular Hematology, Center for Biologics Evaluation and Research, FDA, Bethesda, MD 20892, USA

## Abstract

**Background:**

Previous reports of site-directed deletion analysis on gamma (γ)-phage lysin protein (PlyG) have demonstrated that removal of a short amino acid sequence in the C-terminal region encompassing a 10-amino acid motif (190LKMTADFILQ199) abrogates its binding activity specific to the cell wall of *Bacillus anthracis*. Whether short synthetic peptides representing the10-amino acid PlyG putative binding motif flanked by surrounding N- and C-terminal residues also selectively bind to the bacterial cell wall has not been evaluated. If such peptides do demonstrate selective binding to the cell wall, they could serve as bio-probes towards developing detection technologies for *B. anthracis*. Furthermore, by using *B. anthracis *(Sterne, 34F2), an animal vaccine and *B. cereus*-*4342*, a γ-phage susceptible rare strain as surrogates of *B. anthracis*, development of proof-of-concepts for *B. anthracis *are feasible.

**Results:**

Using four different methods, we evaluated six synthetic peptides representing the putative binding motif including flanking sequences (PlyG-P1 through P6) for the bacterial cell wall binding capacity. Our analysis identified PlyG-P1, PlyG-P3 and PlyG-P5 to have binding capability to both *B. anthracis *(Sterne, 34F2) and *B. cereus-*4342. The peptides however did not bind to *B. cereus*-11778, *B. thuringiensis*, and *B. cereus*-10876 suggesting their specificity for *B. anthracis*-Sterne and *B. cereus*-*4342*. PlyG-P3 in combination with fluorescent light microscopy detected even a single bacterium in plasma spiked with the bacteria.

**Conclusion:**

Overall, these studies illustrate that the short 10-amino acid sequence 'LKMTADFILQ' in fact is a stand-alone bacterial cell wall-binding motif of PlyG. In principle, synthetic peptides PlyG-P1, PlyG-P3 and PlyG-P5, especially PlyG-P3 coupled with Qdot-nanocrystals are useful as high-sensitivity bio-probes in developing detection technologies for *B. anthracis*.

## Background

Spore forms of *Bacillus anthracis *once inhaled, germinate and multiply rapidly in lymph nodes that are in close proximity to lungs. Subsequently the bacteria and its lethal toxin circulate into the blood stream, thereby causing death to the exposed subjects if untreated on time [1-5]. Because of this lethal effect on humans and animals, *B. anthracis *is classified as a category-A bioweapon [6-9]. There are a number of double-stranded DNA bacteriophages that specifically bind, infect and lyse host bacteria through the action of a family of enzymes called lysins that they encode [10-12]. One such enzyme, the γ-phage derived lysin PlyG (Phage lysin-Gamma), was previously shown both *in vitro *and in a Balb/c mouse model to selectively search and kill both *B. anthracis *and a rare variant susceptible to γ-phage, *B. cereus*-4342 [4].

The PlyG like other members of the lysin family contains two domains, an N-terminal catalytic domain and a C-terminal domain that demonstrates high degree of binding specificity to the cell wall peptidoglycans of *B. anthracis *and its nonlethal surrogates [13-15]. Previous analysis of PlyG C-terminal region suggested that a domain spanning residues 156 to 233 of PlyG is sufficient for binding to the *B. anthracis *cell and useful as a probe in detecting the bacteria [16]. Deletion analysis of PlyG 156–233 region further indicated that a PlyG polypeptide lacking amino acids 190 to 199 (LKMTADFILQ) lost its ability to bind to the bacteria, suggesting that this short region imparts binding activity to the PlyG polypeptide [17]. By further mutational analysis, both L190 and Q199 residues of LKMTADFILQ sequence proved to be important for the binding activity of PlyG [17]. In all these studies, larger polypeptides of PlyG served as probes to detect the bacteria. However, whether short synthetic peptides containing LKMTADFILQ amino acid sequence alone can selectively bind to the bacteria with similar specificity or the 10-amino acid sequence imparts cell wall binding capability only in the context of larger PlyG protein is not known. If the former turns out to be true by experimental verification, then such short synthetic peptides will be useful in developing novel detection methods for *B. anthracis *by using its known surrogates, *B. anthracis *(Sterne, 34F2) vaccine strain and another γ-phage susceptible rare bacillus strain, *B. cereus-*4342. The advantage with short synthetic peptides is that large quantities of peptides in pure form can be synthesized. Whereas, achieving purity of larger recombinant proteins often associates with inherent problems such as protein denaturation and misfolding, leading to loss of function [18,19].

In this report, using four different methods, we evaluated six synthetic peptides representing the 10-amino acid PlyG putative binding motif and its variant forms for the bacterial cell wall binding capacity. We successfully identified three synthetic peptides that are effective in selectively binding to *B. cereus*-4342 and *B. anthracis *(Sterne 34F2) in spiked plasma.

## Results

### Synthetic PlyG peptides that include LKMTADFILQ residues demonstrate binding to both *B. cereus*-4342 and vaccine strain of *B. anthracis *(Sterne)

**Figure 1:**
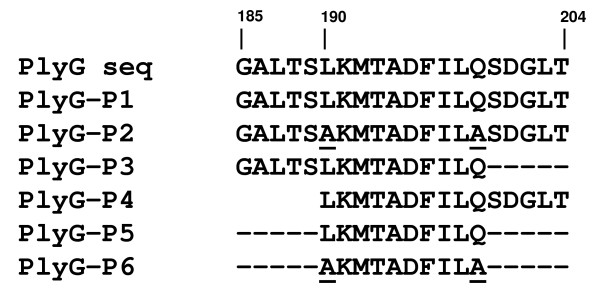
**Schematic representation of PlyG peptides indicating amino acid (aa) position 185–204 of PlyG**. Top sequence Represents PlyG amino acid sequence from 185–204 within the C-terminal 156–233 region. Residues underlined at position 190 and 199 in peptide 2 and 6 indicate amino acid substitutions at that position.

To test whether short synthetic peptide LKMTADFILQ by itself can bind to the cell wall of *B. cereus-*4342 and *B. anthracis *(Sterne), we synthesized six peptides ranging between 10–20-mers within the C-terminal region of PlyG between amino acid positions 185 to 204, which encompass residues 190LKMTADFILQ199 or its variants where L190 and Q199 had substitutions. Figure 1 illustrates the description of each peptide. We examined the binding capacity of these peptides to *B. cereus-*4342, *B. anthracis*-Sterne, *B. cereus*-11778, *B thuringiensis*-10792 and *B. cereus*-10876 by four independent methods: 1. dot-blot assay, 2. ELISA method, 3. fluorometry and 4. Fluorescence-microscopy. In the later two methods, using bacteria-spiked plasma as the detection medium, the peptide bound to bacteria was detected by Qdot-nanocrystal cores conjugated with streptavidin.

### Dot-blot based binding specificity analysis of the peptides

**Figure 2:**
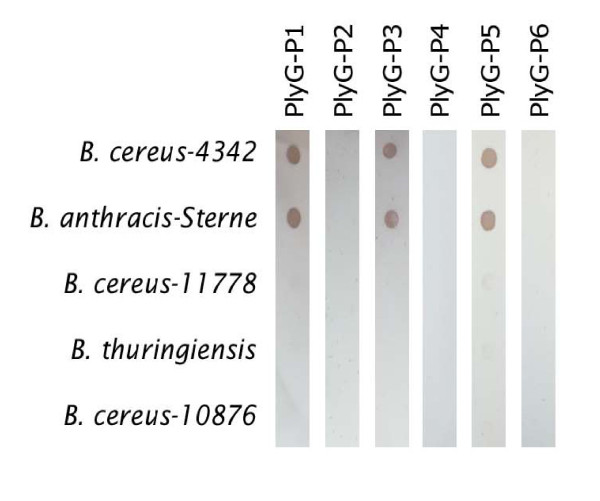
**Dot-blot analysis of peptides binding to *B. cereus*-4342, *B*. *anthracis-*Sterne, *B. cereus*-11778, *B. thuringiensis*, and *B. cereus*-10876**. Bacterial suspensions (10^3 ^CFU) were blotted on to the membrane and the membrane was cut in to strips. The membrane strips were incubated individually with the six PlyG peptides. Following streptavidin-HRP incubation, the membranes were incubated with diaminobenzidine (DAB) reagent and peptide binding was detected by development of brown spots.

Our results revealed that peptides PlyG-P1, P3, and P5 selectively bind to *B. cereus-*4342 and *B. anthracis*-Sterne in the dot-blot assay while peptides PlyG-P2, P4 and P6 did not do so (Figure 2). None of the six peptides bound to *B. cereus-*11778, *B. thuringiensis *and *B. cereus-*10876, although these strains belong to *Bacillus *group, which clearly demonstrates the binding specificity of the PlyG-based peptides for *B. anthracis*-Sterne and *B. cereus*-4342.

### ELISA based binding analysis of the peptides

**Figure 3:**
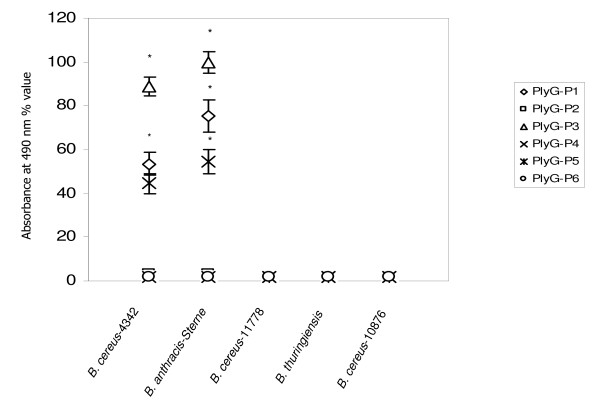
**ELISA-based analysis of PlyG peptide binding to *B. cereus-*4342, *B. anthracis*-Sterne, *B. cereus*-11778, *B. thuringiensis*, and *B. cereus*-10876**. Individual wells of a 96-well microplate were coated with desired bacterial suspensions (10^3 ^CFU) and incubated with biotinylated PlyG peptides prior to incubation with streptavidin-HRP. Color was developed by adding TMB. Optical density of the reactions were measured at 490 nm using a microplate reader. Error bars indicate the SD from three independent experiments. Statistically significant difference (*P *<*0.05*) is represented by (*).

Since PlyG peptides demonstrated specific binding to both *B. cereus-*4342 and *B. anthracis*-Sterne in the dot blot assay, we further evaluated the binding ability of these peptides to the bacteria in an ELISA based assay. Results of this assay method illustrated that peptide PlyG-P1 and PlyG-P3 are strong binders (P < 0.05) to *B. cereus-*4342 and *B. anthracis*-Sterne while PlyG-P5 has relatively lower binding; binding of rest of the peptides including the mutant forms were below the detection level (Figure 3). In this assay, none of the six peptides demonstrated binding to *B. cereus-*11778, *B. thuringiensis *and *B. cereus-*10876 as is the case with the dot-blot method. Statistical evaluation of the ELISA results revealed that binding of PlyG-P1, PlyG-P3 and PlyG-P5 to *B. cereus-*4342 and *B. anthracis*-Sterne was significantly higher (P < 0.05) than the binding of PlyG-P2, PlyG-P4 and PlyG-P6 to the same bacteria.

### Streptavidin-conjugated Quantum Dot (QD)-based binding specificity analysis of the peptides

**Figure 4:**
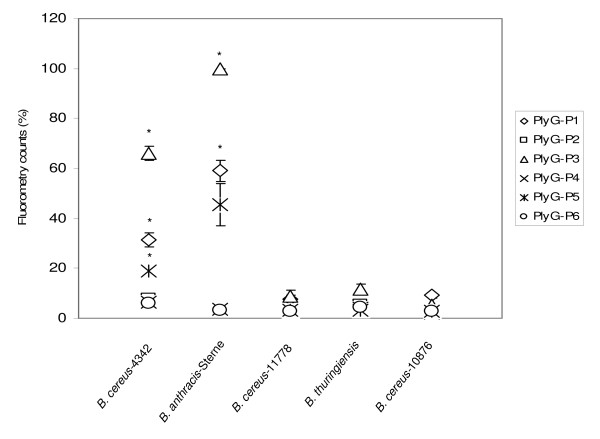
**Streptavidin-conjugated Quantum Dot (QD) based analysis of PlyG peptides binding to bacteria**. Human plasma was spiked with *B. cereus-*4342, *B. anthracis*-Sterne, *B. cereus*-11778, *B. thuringiensis *and *B. cereus*-10876. Each PlyG peptide (P1-P6) were incubated with the bacteria. Following incubation, the samples were centrifuged and resuspended in PBS. Streptavidin-conjugated quantum-dots (QDs) were then added to the sample (bacteria-peptide complex) and analyzed by a fluorescence plate reader (Synergy 4TM, Biotek, USA) with excitation spectrum set at 360–485 nm and emission spectrum at 605 nm. Error bars indicate the SD from 3 independent experiments. Statistically significant difference *(P < 0.05*) is represented by (*).

All six PlyG peptides (P1-P6) were incubated individually with *B. cereus-*4342, *B. anthracis*-Sterne, *B. cereus*-11778, *B. thuringiensis*, and *B. cereus*-10876 cultures and binding of each peptide to the bacteria was detected using Qdot-nanocrystal cores conjugated with streptavidin. Analysis of human plasma spiked with the bacterial suspensions in a micro-well plate was performed using a fluorescence plate reader (Synergy 4™, Biotek, USA). Excitation was set to a spectral range of 360–485 nm and emission was at 605 nm. Fluorometric analysis revealed that in this experimental setting, PlyG-P1, PlyG-P3 and PlyG-P5 were able to bind to *B. cereus-*4342 and *B. anthracis*-Sterne as indicated by the significantly higher levels of fluorometric counts (P < 0.05) compared to the samples containing PlyG-P2, P4 and P6. The analysis also confirmed that binding of peptides PlyG-P1, PlyG-P3 and PlyG-P5 to *B. cereus-*4342 and *B. anthracis*-Sterne was highly specific, as they did not cross-react with *B. cereus*-11778, *B. thuringiensis*, and *B. cereus*-10876 (Figure 4).

### Fluorescence microscopy combined with QD-based binding sensitivity and specificity analysis of PlyG-P3

**Figure 5:**
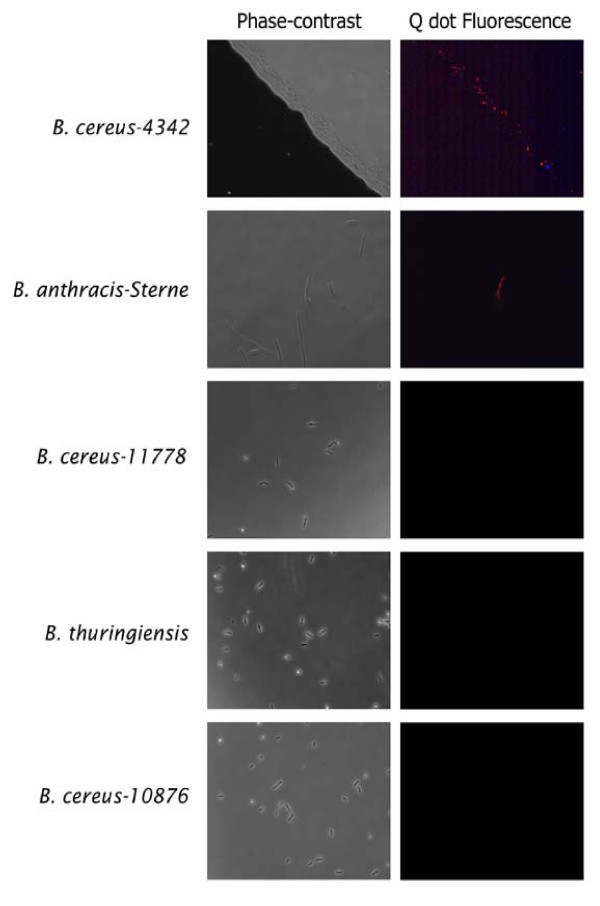
**Microscopy-based sensitivity analysis of PlyG-P3 peptide binding**. Bacterial cultures of *Bacillus cereus *4342, *B. anthracis*-Sterne, *B. cereus*-11778, *B. thuringiensis*, and *B. cereus*-10876 were incubated with PlyG-P3 and the complex was detected by incubating with strepatividin-conjugated quantum dots. Peptide binding was detected by the fluorescing Q dots using appropriate UV filters (605 nm) under a Nikon microscope, either in phase-contrast (panels on left) or fluorescence (panels on right) fields. Note that even a single bacterium is visible in the *B. cereus-Sterne *field.

Experiments so far described in this report indicated that among the six peptides evaluated, PlyG-P3 was the best binder with significantly higher binding (P < 0.05) compared to others. Therefore, we further attempted to confirm this peptide's binding and the limit of detection by a visual method such as fluorescence microscopy. Briefly, human plasma samples spiked with ten-fold dilutions of *Bacillus *cultures were incubated with PlyG-P3 peptide for each dilution. Following incubation, the bacteria-peptide complex was detected with the Qdots under a fluorescence microscope (Nikon Eclipse TE2000-U, USA). The analysis clearly identified that PlyG-P3 peptide specifically binds to the outer surface of *B. cereus-*4342 and *B. anthracis*-Sterne bacterium, providing visual evidence that PlyG-P3 peptide in combination with the Qdot labeling method clearly enhances the detection sensitivity to a single bacterium in the spiked plasma (Figure 5).

## Discussion

In this study, our goal was to refine the previously known *B. anthracis *cell wall binding C-terminal 156–233 region of γ-phage lysin PlyG by employing the minimal sequence as a synthetic peptide that would be useful as a bioprobe for the detection of *B. anthracis*. We have utilized an animal vaccine *B. anthracis *(Sterne) and a rare γ-phage susceptible *B. cereus-*4342 as surrogates for the strain, *B. anthracis *with respect to the cell wall binding ability of the synthetic peptides. In this analysis, we have also included other bacillus members, *B. cereus-*11778, *B. thuringiensis *and *B. cereus-*10876 to serve as controls to illustrate the binding specificity of the synthetic peptides to the two surrogates. Site-directed deletion analysis of PlyG in the past has demonstrated that deletion of a short amino acid sequence in the C-terminal region encompassing a 10-amino acid motif (190LKMTADFILQ199) abrogated its binding activity targeted to the cell wall of *Bacillus anthracis*. However, whether short synthetic peptides containing the 10-amino acid putative binding motif flanked by surrounding N- and C-terminal residues also selectively bind to the bacterial cell wall has not been evaluated.

As a first step, we synthesized six peptides derived from the PlyG lysin polypeptide sequence encompassing the 185GALTSLKMTADFILQSDGLT204 amino acid region [17]. The synthetic peptides were analyzed for their bacterial cell binding capability by using two surrogate strains in spiked plasma as a model for *B. anthracis *detection under bio-safety level 2 (BSL2) conditions. Previously it was shown that PlyG protein bound to both *B. cereus-*4342 and *B. anthracis *with equal affinity due to the identical nature of the cell wall surface as both these bacteria are the monomorphic isolates within the *B. cereus *lineage [4,20,21].

Our results clearly show that the 20-aa peptide PlyG-P1 (185GALTSLKMTADFILQSDGLT204), the 15-aa PlyG-P3 (185GALTSLKMTADFILQ199) and the 10-aa PlyG-P5 (190LKMTADFILQ199) consistently demonstrated selective binding to *B. cereus-*4342 and *B. anthracis-*Sterne in the membrane based dot-blot method (detection level 10^3 ^CFU/ml). Though all three peptides were able to show reactivity to the bacteria, the membrane assay does not reveal the quantitative nature of this binding. Therefore, this estimation was achieved by the ELISA and Fluorometry assays wherein it was further confirmed that the PlyG-P1, PlyG-P3 and PlyG-P5 not only bound very efficiently to *B. cereus-*4342 and *B*. anthracis-Sterne but in addition there was a noticeable quantitative difference between their binding capacity as well. Among the three peptides, PlyG-P3 binding was significantly higher (P < 0.05) than PlyG-P1 and PlyG-P5. The inability of the mutant peptides PlyG-P2 (185GALTS**A**KMTADFIL**A**SDGLT204) and PlyG-P6 (190**A**KMTADFIL**A**199) binding to *B. cereus*-4342 reaffirms a previous report that the L190 and Q199 residues within the binding motif 190LKMTADFILQ199 are critical in order for the larger PlyG protein to bind to the bacterial cell wall [17]. However, it is interesting to note that PlyG-P4 (190LKMTADFILQSDGLT204) lacking five residues 185GALTS189 also demonstrated its inability to bind to *B. cereus-*4342 and *B. anthracis*-Sterne. This result suggest that while these five residues were not identified to be necessary for the binding of recombinant PlyG 156–223 protein to the bacteria in a previous report [17], in the context of shorter synthetic peptide binding ability to the bacteria, addition of these five residues to the N-terminus of 190LKMTADFILQ199 sequence positively contributed to the binding activity. This further suggests that while 190LKMTADFILQ199 region (represented by PlyG-P5 in this study) is a deemed essential motif of lysin PlyG for its binding to the host cell wall, a peptide containing additional five residues on the N-terminal side of this minimum motif seems to be a better peptide for bioprobe development. Fluorometric analysis indicated that PlyG-P1 and PlyG-P3 were the strongest binders to *B. cereus-*4342 *and B. anthracis*-Sterne. When combined with Qdots, the detection sensitivity of PlyG-P3 was enhanced to a single bacterium in the spiked plasma by florescence-microscopy. These findings are encouraging and open up novel strategies in the usage of some of these peptides coupled with Qdots in developing diagnostics for *B. anthracis*.

## Conclusion

Our analysis of PlyG-derived synthetic peptides suggest that three peptides (PlyG-P1, P3 and P5) selectively bound to *B. cereus*-4342 and *B. anthracis*-Sterne, suggesting the validity of using these peptides as bioprobes for the detection of the two strains that serve as surrogates for *B. anthracis *with respect to their cell wall binding capacity. In addition, we identified a self-sufficient stand-alone bacterial cell-binding domain derived from the PlyG protein to be useful as a bio-probe. The sensitivity of detection can be enhanced by using Qdots in combination with the peptides.

## Methods

### Cells, Reagents and Culture

The *Bacillus cereus *strain (RSVF1 strain 4342), *Bacillus cereus *ATCC 11778, *Bacillus cereus *ATCC10876, and *Bacillus thuringiensis *ATCC 10792 were procured from the American Type Culture Collection (ATCC, Manassas, VA). Animal vaccine strain of *B. anthracis *(Sterne 34F2) originally obtained from Colorado Serum Co. (Denver, CO) was a generous gift from Dr. Duncan, CBER, FDA. We utilized brain-heart infusion (BHI) broth (Becton Dickinson, Sparks, MD) for culturing this strain and Miller's Luria Bertani (LB) broth (Mediatech Inc, Herndon, VA) for others. All bacterial cultures were grown at 37°C.

### Synthesis of PlyG peptides

Six varying lengths of peptides (named PlyG-P1 through PlyG-P6) and ranging from 10 to 20 residues, representing amino acid position 185 to 204 encompassing the cell wall binding essential domain of wild type PlyG and its mutant forms were synthesized. The amino acid sequences of the peptides were based on the previously published amino acid sequence of PlyG C-terminal region [17]. The peptides were synthesized at our Core Facility in CBER, FDA, biotinylated with a C6-linker and purified by High Pressure Liquid Chromatography (HPLC) for detection using a horse radish peroxidase (HRP)-conjugate (Figure 1). Peptides were reconstituted in 100 mM NaPO4 and 150 mM NaCl, pH 7.2 buffer at room temperature to a final concentration of 1 M. This stock solution was diluted as needed for further use. We confirmed that all six peptides to have the biotin linkers and are recognized by steptavidin-HRP conjugate, prior to initiating the binding assays.

### Dot blot assay

5-ml early-log-phase culture of *B. cereus*-4342, *B. cereus *ATCC 11778, *B. cereus *ATCC 10876, and *B. thuringiensis *ATCC 10792 were grown in LB broth and *B. anthracis*-Sterne was grown in BHI broth for 3 hr followed by centrifugation at 3,000 × g. The cell pellet was resuspended and 10-fold serial dilutions were made in 1× PBS (pH 7.4, Mediatech Inc, Herndon, VA). Bacterial suspension (10^3 ^CFU/ml) was spotted directly onto a nitrocellulose membrane essentially as described [16]. The membrane was incubated in 5% bovine serum albumin (BSA) solution (Sigma, St. Louis, MO) for 2 h to eliminate non-specific signals on the membrane. Each membrane was incubated separately with one peptide at a final concentration of 1.5 mM. Subsequently the membranes were washed three times with TBST buffer (10 mM Tris-HCl, pH 7.5, 150 mM NaCl, 0.05% Tween-20 (Aldrich Chemical Company, Milwaukee, WI). The membranes were then incubated with 1:10,000 diluted streptavidin-HRP conjugate (Upstate, Temecula, CA) for 60 min at RT and washed three times with TBST buffer. Following washing, the membrane was developed using a DAB (3, 3'- diaminobenzidine) substrate kit (Zymed Laboratories, Carlsbad, CA). The development of colored spots in locations where the bacteria were spotted on the membrane was inferred as positive for peptide binding. The membranes were photographed for archiving the data.

### Enzyme-Linked ImmunoSorbent Assay (ELISA)

The assay was carried out as described previously [22] with modifications. Briefly, 5-ml of early-log-phase bacterial cultures of *B. cereus*-4342, *B. anthracis*-Sterne, *B. cereus*-11778, *B. cereus*-10876, and *B. thuringiensis *were centrifuged at 3,000 × g. The cell pellet was resuspended and 10-fold serial dilutions were made in 1× PBS. Bacterial cell suspension at a concentration of 10^3 ^CFU was then layered into the wells of 96-well micro plate (Becton Dickinson, Bedford, MA) and incubated overnight at RT. Subsequently, cells were fixed in ethanol (Aldrich Chemical Company, Milwaukee, WI) for 10 min and the plates were air-dried. Wells were blocked with 5% BSA (Sigma, St. Louis, MO) for 60 min at RT, rinsed with PBS and then PlyG peptides suspended in PBS at a final concentration of 10 mM were added to all the wells and incubated for 15 min. Following incubation, wells were washed 3 times with PBST buffer (PBS pH 7.4, 0.01% Tween 20). The wells were further incubated with 1:10,000 dilution of streptavidin-HRP conjugate (Upstate, Temecula, CA) for 15 min and washed with PBST buffer. Tetramethylbenzidine (TMB) membrane peroxidase substrate (Zymed Laboratories, Carlsbad, CA) system was used to detect the enzyme label in accordance with the manufacturer's instructions. The color development in the 96-well plate was recorded by using Synergy 4™ BioTek micro plate reader (BioTek Instruments, Winooski, VT) at 490 nm wavelength. Assays were repeated three times for statistical analysis.

### Q-dot based fluorescence assay

Log-phase cultures of *B. cereus*-4342, *B. anthracis*-Sterne, *B. cereus*-11778, *B. cereus*-10876, and *B. thuringiensis *were subjected to low-speed centrifugation and the pellet was resuspended in 1 × PBS. Ten-fold serial dilutions were each made in 1 ml of human plasma [23] and 10^3 ^CFU/ml was used for spiking the plasma. The rationale for spiking 10^3 ^CFU/ml of bacteria was based on the consensus in the field, which suggests that the limit of detection for commercially available rapid but less sensitive bacterial detection systems is 10^3^–10^5 ^CFU/ml (). Peptide binding and detection assay was performed in a final volume of 100 μl in an eppendorf tube kept at RT for 90 min. Following the binding step, tubes were centrifuged for 5 minutes at 3,000 × g and the pellets were washed with PBS and resuspended in 100 μl of 1:10,000 diluted streptavidin-conjugated Q dots (QD 605) solution (Invitrogen, Gaithersberg, MD). After incubating for 90 min at RT, samples were centrifuged again as above and the pellets were resuspended in 150 μl of PBS. Samples were then placed on a glass slide (Mercedes Medical, Sarasota, FL) or a micro well plate (Becton Dickinson Lab ware, Bedford, MA) and analyzed either under a fluorescence microscope [24] using Nikon Eclipse TE2000-U (Nikon Instruments, Melville, NY) or by fluorometry [25] using Synergy 4™ BioTek micro plate reader (BioTek Instruments, Winooski, VT) respectively. Images as visualized under the microscope were captured by an intensified cooled charge-coupled device-equipped camera (Nikon Eclipse TE2000-U).

### Statistical analyses

We performed all assays described here in triplicates. Mean values ± SD (Standard Deviation) were calculated using Microsoft Excel^®^. Statistical analyses were performed using Student's *t *test and values were considered significant when *P*< 0.05.

## Competing interests

The authors declare that they have no competing interests.

## Authors' contributions

SS and KVKM planned, performed the experiments and wrote the manuscript. CA as Principal Investigator of this project conceived the idea, supervised the staff, and wrote and copyedited the manuscript. All authors read and approved the final manuscript.
